# Epidemiological shifts and temporal trends in emergency department anaphylaxis management: An 18-year longitudinal study in northern Thailand^☆☆^

**DOI:** 10.1016/j.waojou.2026.101460

**Published:** 2026-09-04

**Authors:** Pimchanok Rongdet, Pongsathorn Saligupta, Mongkol Lao-Araya

**Affiliations:** Department of Pediatrics, Faculty of Medicine, Chiang Mai University, Chiang Mai, 50200, Thailand

**Keywords:** Allergic reactions, Epidemiological transition, Etiology, Food allergy, Epinephrine

## Abstract

**Background:**

Although anaphylaxis incidence is rising globally, long-term longitudinal data from Southeast Asia remain scarce. This study evaluates 18-year trends in anaphylaxis visit rates, etiological triggers, and clinical management in Northern Thailand, specifically assessing the impact of institutional protocols implemented in alignment with the 2017 Thai National Clinical Practice Guidelines for Anaphylaxis.

**Methods:**

We conducted an 8-year retrospective study (2017–2024) at a tertiary university hospital, integrating data with a previous 10-year cohort (2007–2016) under an identical clinical adjudication and exclusion protocol. Cases were identified via ICD-10 codes and manually adjudicated using 2006 NIAID-FAAN clinical criteria. We analyzed demographics, triggers, management metrics, and outcomes using multivariable logistic regression. Longitudinal trends adjusted for annual Emergency Department (ED) volumes were evaluated using a Poisson log-linear regression model with a log offset.

**Results:**

Among 647 anaphylaxis events (626 patients), the 2017–2024 presentation rate was 360.7/100,000 ED visits in children and 282.4/100,000 in adults. Adjusting for ED volume, Poisson regression revealed a compounding yearly rate increase of 10% in adults (IRR = 1.10, 95% CI: 1.09–1.12, p<0.001), while pediatric rates remained volatile without a longitudinal trend (IRR = 1.01, 95% CI: 0.98–1.05, p=0.40). Total institutional encounters rose significantly (IRR = 1.09, 95% CI: 1.08–1.10, p<0.001). Comparing to the previous 2007–2016 data, food led triggers (49.6%), with growing proportions of shellfish (34.2%) and edible insects (10.5%, p<0.001). Medications caused 20.2% of cases, whereas Hymenoptera stings (10.2%) decreased significantly (p<0.001). Primary manifestations were cutaneous (95.4%), respiratory (66.8%), and gastrointestinal (53.3%). Over the protocol integration timeline, ED intramuscular epinephrine administration rose from 87.5% to 98.3% (p<0.001), and mean door-to-treatment time dropped from 16.3 to 10.3 min (p=0.03). Fatality was zero; however, the discharge prescription rates for self-injectable epinephrine remained stagnant at 7.6%.

**Conclusions:**

Anaphylaxis ED visit rates demonstrated a significant longitudinal increase over 18 years, driven by the adult population and characterized by an evolving distribution of food triggers toward shellfish and edible insects.

## Introduction

Anaphylaxis is an acute, multisystemic, and potentially fatal hypersensitivity reaction characterized by the rapid onset of life-threatening respiratory or circulatory compromise. Given its unpredictable and often progressive nature, the condition demands immediate clinical recognition and prompt medical intervention.^1^^,^^2^ Over the last 2 decades, global epidemiological data have documented a significant rise in anaphylaxis incidence.^3^^,^^4^ Although mortality rates have remained relatively stable, this increasing prevalence has placed a substantial burden on emergency departments and healthcare systems worldwide.^1^^,^^3^^,^^4^

The triggers and clinical presentation of anaphylaxis exhibit significant geographic heterogeneity, shaped by regional dietary patterns, environmental exposures, and local medical practices.^3^ While food and insect venom are the primary drivers in Western nations,^5^^,^^6^ Asian cohorts have historically identified medications and specific local food allergens as predominant causes.^7^ However, recent evidence suggests an epidemiological transition in Asia, with food-induced anaphylaxis becoming increasingly prevalent—a shift that mirrors patterns observed in Western countries.^8^^,^^9^ Understanding these regional nuances is essential for refining prevention strategies and developing clinical guidelines tailored to specific populations.

In Thailand, although several cross-sectional studies have characterized anaphylaxis,^10^, ^11^, ^12^ there remains a paucity of long-term longitudinal data. Northern Thailand, served by Chiang Mai University Hospital (CMU) Hospital—a prominent tertiary referral center—offers a unique clinical landscape for studying these trends. Our previous retrospective analysis at CMU Hospital (2007–2016) identified an annual anaphylaxis occurrence of 93.8 episodes per 100,000 emergency visits, with food (47%), Hymenoptera stings (23%), and medications (18%) as the primary culprits.^10^ Following the publication of those findings, hospital-wide best practice guidelines for anaphylaxis care were developed and implemented at our institution. In the years since, shifting lifestyles, the introduction of novel pharmaceutical agents, and heightened diagnostic vigilance—potentially influenced by the implementation of these new protocols—have likely transformed the regional epidemiology of the condition.

To capture these evolving patterns, we conducted an 8-year retrospective analysis of anaphylaxis cases at CMU Hospital spanning 2017 to 2024. The primary objective of this study was to evaluate the shifting trends in incidence, etiological triggers, and clinical characteristics over this period. By integrating these findings with our previously published data (2007–2016),^10^ we provide a comprehensive 18-year longitudinal overview (2007–2024). We aim to offer evidence-based clinical insights that will optimize acute management, resource allocation, and long-term preventive care for patients at risk of anaphylaxis in Northern Thailand.

## Materials and methods

### Study setting and population

This retrospective analysis was conducted at CMU Hospital in Chiang Mai, Thailand. As a tertiary care medical center with 1100 beds, CMU Hospital serves as the primary emergency facility for approximately 235,000 urban residents and acts as the tertiary referral hub for over 11 million people across Northern Thailand. The facility manages roughly 35,000 emergency visits and 1.4 million outpatient visits annually. While medical services are readily accessible within the Chiang Mai urban area, milder emergency events may occasionally be managed at nearby primary care units or private hospitals.

Potential cases were identified by screening electronic medical records (EMR) for outpatient and emergency department (ED) visits between January 2017 and December 2024. We integrated data from 2 consecutive periods: a previously published cohort (2007–2016)^10^ and a new cohort (2017–2024) to provide an 18-year trend analysis. To ensure absolute data homogeneity and comparability between the 2 cohorts, the historical dataset was completely re-audited; the same EMR diagnostic search architecture, identical exclusion terms, and the same clinical evaluation parameters were uniformly applied across all 18 years.

Potential cases were identified by screening the EMR for the following ICD-10 codes: T78.0 (Anaphylactic shock due to adverse food reaction), T78.2 (Anaphylactic shock, unspecified), T80.5 (Anaphylactic shock due to serum), T88.6 (Anaphylactic shock due to adverse drug effect). Following the initial screening, diagnosis was manually adjudicated and confirmed according to the 2006 National Institute of Allergy and Infectious Diseases/Food Allergy and Anaphylaxis Network (NIAID-FAAN) symposium clinical criteria.^13^ The 2006 consensus criteria were intentionally maintained across the entire 18-year analysis window to preserve diagnostic consistency and eliminate classification drift over time. To be included in the final analysis, patients were required to meet the following inclusion criteria: (1) presentation to the ED or outpatient clinics between January 2017 and December 2024 with acute-onset symptoms, and (2) clinical confirmation of anaphylaxis based on the aforementioned criteria. “Acute onset' was operationalized as the development of symptoms within minutes up to several hours following exposure to a suspected allergen. To ensure a homogenous cohort of community-acquired, out-patient onset presentations, we applied strict exclusion criteria. We excluded inpatient-onset anaphylactic events and patients referred from external facilities solely for elective allergy investigations or follow-up care. However, acute reactions occurring in supervised day units (eg, outpatient chemotherapy suites or imaging units) were included if they necessitated an immediate ED response call. The exclusion criteria (eg, duplicate visits, localized reactions, or incomplete clinical records) were retroactively and uniformly applied to the historical dataset. To ensure data homogeneity across the entire 18-year study period, the inclusion and exclusion criteria, including the systematic omission of duplicate visits, localized hypersensitivity reactions, and incomplete clinical records, were uniformly and retroactively applied to the historical cohort (2007–2016) in exact alignment with the protocol utilized for the contemporary cohort (2017–2024). Standardized data collection form was utilized to extract information from medical charts. Collected variables included demographics such as age, sex, and underlying comorbidities; allergy history including atopic status and previous hypersensitivity reactions; clinical presentation, specifically involving symptoms, the time interval between allergen exposure and symptom onset, and treatment interventions; and clinical outcomes, including response to therapy and final patient disposition.

Participants aged under 15 years were categorized as the “children” group. Anaphylaxis severity was graded based on the established Brown Grading System:^14^ cases presenting with visceral or multi-system involvement without immediate physiologic failure were classified as “mild-to-moderate' (Grades 1–2), whereas cases presenting with immediate organ compromise (hypoxia <92% on room air, cyanosis, hypotension, or loss of consciousness) were classified as “severe' (Grade 3). Pediatric tachycardia was evaluated using age-stratified physiologic vital sign cut-offs (>160 bpm for age <1 year, >140 bpm for 1–3 years, >120 bpm for 4–11 years, and >100 bpm for age over 11 years) to avoid adult-normative over ascertainment. Etiological triggers were determined via a comprehensive review of medical records by an allergist. Cases involving dishes containing multiple hidden or mixed food elements where a single raw allergen could not be clinically isolated were explicitly designated as “Complex Foods”. Due to institutional resource limitations, secondary confirmatory investigations—including skin prick testing, serum-specific IgE assays, or controlled provocation challenges—were performed only in a select subset of the study population.

The study protocol received formal approval from the Research Ethics Committee of the Faculty of Medicine, Chiang Mai University (PED-2568-0819), ensuring compliance with ethical standards for retrospective data analysis.

### Statistics

All statistical analyses were performed using IBM SPSS Statistics for Windows, version 23.0 (IBM Corp., Armonk, NY, USA). Descriptive statistics were used to characterize the study population. Normally distributed continuous variables are expressed as mean ± standard deviation (SD), whereas non-normally distributed continuous variable is presented as median and interquartile range (IQR). Categorical variables are expressed as absolute frequencies and percentages. Between-group comparisons were conducted using the Student's t-test or Mann-Whitney *U* test for continuous data, while the chi-squared test or Fisher's exact test was employed for categorical variables. To identify potential factors associated with severe anaphylaxis, univariate and multivariable logistic regression analyses were performed. Candidate variables with p < 0.20 in the univariate analysis were included in the multivariable model. Results are presented as odds ratio (OR) with 95% confidence interval (95% CI).

Longitudinal trends in anaphylaxis ED visitation rates were evaluated using 2007 as the reference baseline year via a Poisson log-linear regression model, formulated as:

$$\log\left(E\left[Y_{t}\right]\right)=\beta_{0}+\beta_{1}\cdot {\text{Year}}_{t}+\log\left({\text{ED}\;\text{Visits}}_{t}\right)$$

where $Y_{t}$ represents the annual anaphylaxis case count in year t, $\beta_{0}$ is the intercept, $\beta_{1}$ denotes the annual log-rate change, and $\log\left({\text{ED}\;\text{Visits}}_{t}\right)$ serves as a structural offset to account for temporal variations in total annual ED utilization. To evaluate categorical shifts between the 2 study periods (2007–2016 vs. 2017–2024), baseline characteristics and proportions were compared using Pearson's chi-squared test or Fisher's exact test, as appropriate. Furthermore, the structural disruption of the COVID-19 pandemic on ED volumes (2020–2021) was separately evaluated using non-parametric Mann-Whitney U tests. For all statistical tests, a two-sided P-value <0.05 was considered statistically significant.

## Results

During the 8-year study period (2017–2024), CMU Hospital recorded a total of 7,450,880 outpatient visits (adults: 6,101,305; children: 946,279) and 224,410 ED visits (adults: 206,105; children: 18,305). Initial screening using the specified ICD-10 codes identified 1156 visits involving 867 unique patients. Of these, 509 visits (44.0%) were excluded for the following reasons: follow-up episodes rather than acute presentations, referrals from other facilities for elective allergy investigations, inpatient-onset events, or failure to meet the NIAID-FAAN clinical diagnostic criteria. Ultimately, 647 anaphylactic events involving 626 patients met the inclusion criteria and were included in the final analysis. Between 2017 and 2024, the mean anaphylaxis ED visit rate at CMU Hospital was 288.3 episodes per 100,000 ED visits. Age-stratified rates were 282.4 and 360.7 episodes per 100,000 ED visits in adults and children, respectively. The annual distribution of ED volumes and crude anaphylaxis rate are detailed in Table 1. Compared to the historical baseline period (2007–2016), the annual rates during 2017–2024 demonstrated a significant upward trajectory. Poisson log-linear regression models, adjusted for annual encounter volumes, demonstrated that the adult anaphylaxis visitation rate expanded by 10.17% annually over the entire 18-year timeline (Incidence Rate Ratio [IRR] = 1.1017, 95% CI: 1.0873–1.1162, *p < 0.001*) (Table 2). Conversely, the pediatric presentation rate exhibited marked longitudinal volatility without a monotonic trend (IRR = 1.0144, 95% CI: 0.9811–1.0487, *p* = *0.40*), characterized by a sharp decline in cases in 2022 (89.6 per 100,000 visits) bracketed by pronounced spikes in 2021 (534.1 per 100,000 visits) and 2023 (468.7 per 100,000 visits).

**Table 1:** Crude anaphylaxis rates per 100,000 emergency department (ED) visits and annual ED visits (2007–2024)

	Crude anaphylaxis rates (per 100,000 ED visits)			Annual ED visits (all diseases)		
Year	Children	Adults	Total rate	Children	Adults	Total
2007	250.3	33.6	45.8	1598	26,770	28,368

2008	203.4	96.2	103.3	1967	28,057	30,024

2009	372.2	94.3	119.0	2687	27,564	30,251

2010	511.4	53.8	97.3	2933	27,904	30,837

2011	294.8	102.7	120.1	2714	27,261	29,975

2012	388.7	169.3	189.2	2830	28,348	31,178

2013	144.6	149.0	148.6	2767	28,194	30,961

2014	212.2	179.0	182.1	2827	27,928	30,755

2015	363.9	213.3	226.5	2748	28,597	31,345

2016	69.9	210.5	197.9	2861	28,974	31,835

2017	201.0	227.2	225.0	2488	27,289	29,777

2018	354.3	225.3	236.2	2540	27,523	30,063

2019	303.6	211.9	219.7	2635	28,321	30,956

2020	610.4	228.4	257.2	1966	24,079	26,045

2021	534.1	238.4	260.4	1685	20,972	22,657

2022	89.6	366.1	342.9	2232	24,308	26,540

2023	468.7	387.9	394.5	2347	26,551	28,898

2024	414.6	373.2	376.6	2412	27,062	29,474

**Table 2:** Annual trends and incidence rate ratios (IRR) of emergency department anaphylaxis in pediatric and adult populations (2007–2024).

Patient group	Coefficient (β1)	IRR (e^β1^)	95% CI	p-value
Children	0.0143	1.0144	0.9811–1.0487	0.4019

Adults	0.0969	1.1017	1.0873–1.1162	*<0.0001*

Total	0.086	1.0898	1.0767–1.1030	*<0.0001*


Models evaluated using Poisson log-linear regression with a log offset for total ER visits to adjust for varying underlying patient volumes.

**Fig. 1:**
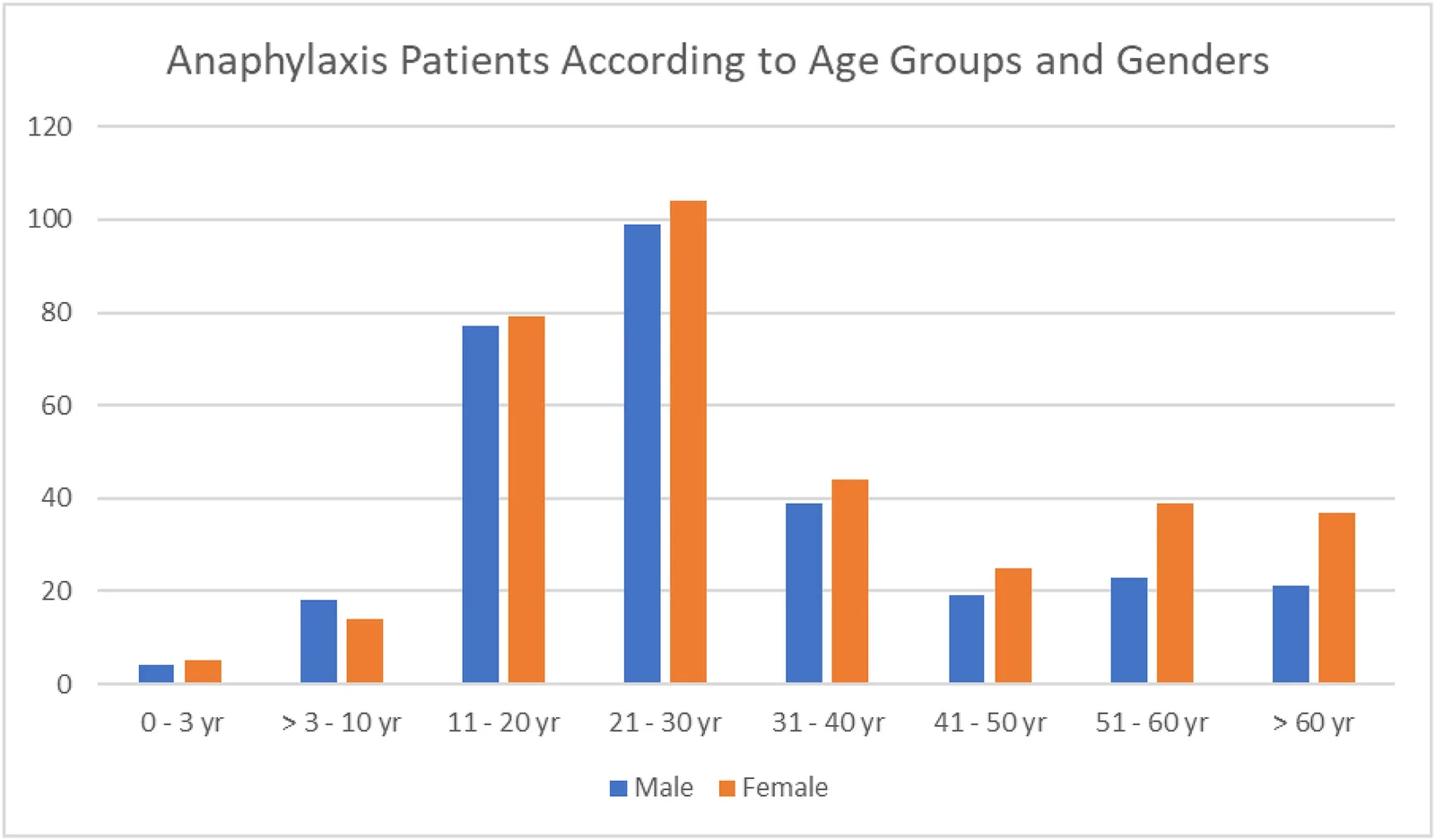
Distribution of anaphylaxis frequency categorized by age group (years) and gender

Importantly, longitudinal trend analyses were heavily influenced by shifts in baseline healthcare utilization. Non-parametric analysis (Mann-Whitney *U* test) revealed that total institutional ED volumes contracted significantly by 20.13% (*p* = *0.02*) during the peak COVID-19 pandemic years (2020–2021) relative to the pre-pandemic baseline (2007–2019). This structural reduction in total encounters arithmetically inflated the crude visitation rates during the pandemic era. The clinical characteristics of the study population are summarized in Table 3. The median age was 10.0 years (IQR: 6.5–14.0) for children, 28.0 years (IQR: 21.0–45.0) for adults, and 25.0 years (IQR: 20.0–41.0) for the overall cohort. Approximately one-third (32.0%) of the patients had a documented history of allergic disease. A history of food allergy was present in 23.0% of the participants, with no significant difference observed between pediatric and adult groups. However, the coexistence of asthma was significantly more frequent in children compared to adults (8.3% vs. 2.6%). Additionally, 53 patients (8.3%) reported a history of prior anaphylactic episodes. When compared with data from the 2007–2016 period,^10^the current cohort (2017–2024) demonstrated a statistically significant reduction in documented comorbid asthma (3.2% vs. 6.5%, p = 0.01) but a higher proportion of patients with a history of recurrent anaphylaxis (8.3% vs 3.2%, p < 0.001). The distribution of anaphylaxis frequency by age group and gender is illustrated in Fig. 1. The highest frequency of anaphylaxis occurred among adolescents and young adults, specifically in the 11–20 and 21–30 age brackets. A male predominance was observed in children aged between 3 and 10 years. Conversely, a female predominance was noted among children under 3 years of age, teenagers, and the adult population.

**Table 3:** Characteristics of patients with anaphylaxis (no. Of patients (%)).

	2017–2024			2007–2016 Total (n = 433)	P-value^a^
	Children (n = 59)	Adult (n = 567)	Total (n = 626)		
Median age (IQR, years)	10.0 (6.5–14.0)	28.0 (21.0–45.0)	25.0 (20.0–41.0)	24.0 (19.0–43.0)	0.88

Sex: Male	30 (50.8)	260 (45.8)	301 (48.1)	202 (46.6)	0.65

History of allergic disease (%)	18 (30.5)	182 (32.1)	200 (32.0)	141 (32.5)	0.83

• Food allergy	13 (22.0)	149 (26.3)	162 (25.9)	99 (22.8)	0.26

• Allergic rhinitis	3 (5.1)	34 (6.0)	37 (5.9)	22 (5.1)	0.56

• Asthma	5 (8.4)^b^	15 (2.6)^b^	20 (3.2)	28 (6.5)	*0.01*

• Atopic dermatitis	1 (1.7)	0 (0)	1 (0.2)	2 (0.5)	0.36

History of recurrent anaphylaxis	4 (6.8)	48 (8.5)	52 (8.3)	14 (3.2)	*<0.001*


a Comparisons between the 2007–2016 and 2017–2024 groups.

b Statistical significance was observed when comparing pediatric and adult groups during the 2017–2024 period (p = 0.02).

The most common triggers of anaphylaxis were foods (49.6%), medications (20.2%), and Hymenoptera stings (10.2%), as detailed in Table 4. The hierarchy of these 3 leading causative agents remained consistent across both pediatric and adult cohorts. When compared with the 2007–2016 study period,^10^ food remained the most frequent trigger in both age groups, with the overall proportion of food-induced anaphylaxis remaining stable. However, the specific composition of food allergens shifted significantly. The rates of anaphylaxis attributed to shellfish and edible insects were statistically higher in the current cohort compared to the previous period (34.2% vs. 25.6%, *p* = *0.003*; and 10.5% vs. 3.9%, *p < 0.001*, respectively). While shrimp remained the most prevalent individual trigger in both cohorts, edible insects—particularly silkworms, weaver ant larvae (ant eggs), and bee broods—emerged as increasingly common allergens. Additionally, 67 patients (10.3%) presented with food-induced anaphylaxis following the consumption of “complex foods"—meals prepared with multiple ingredients where the specific culprit allergen could not be definitively identified.

**Table 4:** Causes of anaphylaxis (no. Of events (%)).

	2017–2024			2007–2016 Total (n = 441)	P-value^a^
	Children (n = 60)	Adult (n = 587)	Total (n = 647)		
**1. Foods**^b^	**33 (55.0)**	**288 (49.1)**	**321 (49.6)**	**209 (47.4)**	**0.52**

**Shellfish**	**21 (35.0)**	**200 (34.1)**	**221 (34.2)**	**113 (25.6)**	***0.003***

• Shrimp	13 (21.7)	132 (22.5)	145 (22.4)	58 (13.2)	

• Crab	3 (5.0)	16 (2.7)	19 (2.9)	18 (4.1)	

• Squid	2 (3.3)	13 (2.2)	15 (2.3)	10 (2.2)	

• Mollusk	3 (5.0)	13 (2.2)	16 (2.4)	NA	

• Unspecified	0	26 (4.4)	26 (4.0)	NA	

Fish	0	7 (1.2)	7 (1.0)	17 (3.9)	

Milk	2 (3.3)	2 (0.3)	4 (0.6)	NA	

Fruits/Vegetables	1 (1.7)	10 (1.7)	11 (1.7)	6 (1.4)	

Wheat/Legumes	1 (1.7)	3 (0.5)	4 (0.6)	1 (0.22)	

Complex food	8 (13.3)	59 (10.1)^c^	67 (10.3)	27 (6.1)	

**Edible insects**	**6 (10.0)**	**62 (10.6)**	**68 (10.5)**	**17 (3.9)**	***<0.001***

• Weaver ant larvae	2 (3.3)	10 (1.7)	12 (1.9)	6 (1.4)	

• Silkworm	1 (1.7)	22 (3.7)	23 (3.5)	NA	

• Bee broods	1 (1.7)	7 (1.2)	8 (1.2)	NA	

• Cricket	0	2 (0.3)	2 (0.3)	NA	

• Others or unidentified insects	2 (3.3)	21 (3.6)	23 (3.6)	11 (2.5)	

**2. Insect stings**	**6 (10.0)**	**60 (10.2)**	**66 (10.2)**	**102 (23.1)**	***<0.001***

Bees	1 (1.7)	18 (3.1)	19 (2.9)	26 (5.9)	

Wasps/hornets	4 (6.7)	15 (2.6)	19 (2.9)	26 (5.9)	

Ants	1 (1.7)	5 (0.9)	6 (0.9)	11 (2.5)	

Unspecified insects	1 (1.7)	21 (3.6)	22 (3.4)	41 (9.3)	

**3. Drugs**	**13 (21.7)**	**125 (21.3)**	**138 (20.2)**	**79 (17.9)**	**0.34**

NSAIDS	1 (1.7)	34 (5.8)	35 (5.4)	33 (7.4)	

• Ibuprofen	1 (1.7)	15 (2.6)	16 (2.5)	13 (2.9)	

• Diclofenac	0	8 (1.4)	8 (1.2)	10 (2.3)	

• Naproxen	0	4 (0.7)	4 (0.6)	10 (2.3)	

• COX-2 inhibitor	0	3 (0.5)	3 (0.4)	NA	

Antimicrobial agents	1 (1.7)	27 (4.6)	28 (4.3)	18 (4.0)	

• Beta-lactam	1 (1.7)	22 (3.7)	23 (3.6)	7 (1.6)	

• Amoxicillin	1 (1.7)	15 (2.6)	16 (2.5)	NA	

• Cephalosporin	0	6 (1.0)	6 (0.9)	NA	

• Quinolone	0	5 (0.9)	5 (0.7)	2 (0.5)	

Paracetamol	1 (1.7)	4 (0.7)	5 (0.7)	0	

Proton pump inhibitor	0	3 (0.5)	3 (0.4)	0	

Contrast media	1 (1.7)	13 (2.2)	14 (2.1)	4 (0.9)	

Chemotherapy	0	2 (0.3)	2 (0.3)	NA	

Herbs	0	2 (0.3)	2 (0.3)	NA	

Unknown	0	9 (1.5)	9 (1.3)	NA	

Others	2 (3.3)	30 (5.1)	32 (4.9)	24 (5.4)	

**4. Other substances**	**0**	**4 (0.7)**	**4 (0.6)**	**5 (1.1)**	**0.85**

Hair dye	0	2 (0.3)	2 (0.3)	NA	

Inhalant allergens	0	2 (0.3)	2 (0.3)	NA	

**5. Idiopathic**	**8 (13.3)**	**49 (8.3)**	**57 (8.8)**	**46 (10.4)**	**0.80**


a Comparisons of proportions between the 2007–2016 and 2017–2024 groups.

b Patients reacting to multiple food items had each culprit allergen counted individually across specific food categories.

c Alcohol was identified as an augmenting cofactor in 2 adult events involving concurrent exposure to multiple foods and medications.

The proportion of anaphylaxis attributed to insect stings decreased significantly compared to the previous period^10^ (10.2% vs. 23.1%, *p < 0.001*). While bees remained the primary causative agent among identifiable Hymenoptera, the specific insect could not be identified during the ED evaluation in nearly half of these cases. The proportion of drug-induced anaphylaxis remained stable between the 2 study periods. Non-steroidal anti-inflammatory drugs (NSAIDs) and antibiotics continued to be the most common pharmacological triggers. Emerging triggers in the current cohort included proton-pump inhibitors (PPIs), herbal supplements, and biological or chemotherapeutic agents. Following the implementation of 2017 institutional guidelines—which required immediate ED transfer for reactions occurring in supervised day units—14 radiocontrast media and 2 chemotherapy-induced anaphylaxis cases were included in this cohort. The clinical manifestations observed upon arrival at the ED are summarized in Table 5. Among the 647 anaphylactic events, the organ systems involved included the cutaneous and mucosal (95.4%), respiratory (66.8%), gastrointestinal (53.3%), cardiovascular (45.0%), and neurological (1.5%) systems. Cardiovascular symptoms, particularly tachycardia, were significantly more prevalent in the pediatric group compared to adults (83.3% vs. 30.3%, *p* = *0.001*). Interestingly, when compared to the 2007–2016 data,^10^ the current cohort demonstrated a higher prevalence of hypotension and gastrointestinal symptoms upon ED arrival. Conversely, alterations in consciousness, including syncope, were less frequently observed in the current study period than in the previous decade.

**Table 5:** Clinical presentations (no. Of events (%)).

	2017–2024			2007–2016 Total (n = 441)	P-value^a^
	Children (n = 60)	Adult (n = 587)	Total (n = 647)		
Skin and mucosa	59 (98.3)	558 (95.1)	617 (95.4)	416 (94.3)	0.45
• Urticaria	55 (91.7)	516 (87.9)	571 (88.3)	371 (84.1)	0.35
• Angioedema	19 (31.7)	132 (22.5)	151 (23.3)	152 (34.5)	*<0.001*
• Eye redness	1 (1.7)	3 (0.5)	4 (0.6)	8 (1.8)	0.06
Respiratory symptoms	38 (63.3)	394 (67.1)	432 (66.8)	299 (67.8)	0.72
• Chest discomfort	17 (28.3)	331 (56.4)	348 (53.8)	257 (58.3)	0.14
• Wheezing	26 (43.3)	110 (18.7)	136 (21.0)	105 (23.8)	0.28
• Rhinitis	4 (6.7)	41 (7.0)	45 (7.0)	5 (1.1)	*<0.001*
• Upper airway obstraction	2 (3.3)	18 (3.1)	20 (3.1)	13 (2.9)	0.89
Cardiovascular symptoms^b^	51 (85.0)^b^	240 (40.9)^b^	291 (45.0)	239 (54.2)	*0.005*
• Hypotension^b^	10 (16.7)^b^	153 (26.1)^b^	163 (25.2)	80 (18.1)	*0.006*
• Tachycardia^b^	50 (83.3)^b^	178 (30.3)^b^	228 (35.2)	199 (45.1)	*0.001*
• Syncope	0	23 (3.9)	23 (3.6)	17 (3.7)	0.80
Gastrointestinal symptoms	32 (53.3)	313 (53.3)	345 (53.3)	194 (44.0)	*0.004*
• Diarrhea	9 (15.0)	159 (27.1)	168 (26.0)	81 (18.4)	*0.003*
• Nausea/Vomiting	24 (40.0)	186 (31.7)	210 (32.5)	112 (25.4)	*0.012*
• Abdominal pain	12 (20.0)	140 (23.9)	140 (21.6)	59 (13.4)	*<0.001*
Neurological symptoms	1 (1.7)	9 (1.5)	10 (1.5)	27 (7.4)	*<0.001*
• Alteration of consciousness	1 (1.7)	9 (1.5)	10 (1.5)	24 (6.4)	*<0.001*
Respiratory failure	0	1 (0.2)	1 (0.2)	2 (0.5)	0.33
Death	0	0	0	0	NS

a Comparisons of proportions between the 2007–2016 and 2017–2024 groups.

b Statistical significance was observed when comparing pediatric and adult groups during the 2017–2024 period (p < 0.05).

The management and clinical outcomes of anaphylaxis are summarized in Table 6. Nearly all patients (98.3%) received intramuscular epinephrine, representing a significantly higher administration rate compared to the 2007–2016 period^10^ (87.5%, *p < 0.001*). Regarding adjunctive therapies, the use of systemic corticosteroids, antihistamines, and intravenous fluids was more frequent in the current cohort. Conversely, the administration of nebulized beta-agonists and supplemental oxygen was less common.

**Table 6:** Treatment and outcomes of anaphylaxis (no. Of events (%)).

	2017–2024			2007–2016 Total (n = 441)	P-value^a^
	Children (n = 60)	Adult (n = 587)	Total (n = 647)		
Time from onset of symptoms to ER arrival					
• <30 min	12 (20.0)	82 (14.0)	94 (14.5)	215 (48.8)	<0.001
• 30–60 min	16 (26.7)	163 (27.8)	179 (27.7)	110 (24.9)	0.32
• > 60 min	32 (53.3)	342 (58.3)	374 (57.8)	116 (26.3)	<0.001
Mean time from ED arrival to first dose of epinephrine (minutes ± S.D.)	12.3 ± 5.4^b^	10.1 ± 8.1^b^	10.3 ± 7.5	16.3 ± 6.7	0.03
**Treatment**					
• Epinephrine IM	60 (100)	576 (98.1)	636 (98.3)	386 (87.5)	<0.001
• Antihistamine	59 (98.3)	575 (98.0)	634 (98.0)	397 (90.0)	<0.001
Systemic corticosteroid	59 (98.3)	567 (96.6)	626 (96.8)	394 (89.3)	<0.001
Nebulized beta-agonist	21 (35.0)^b^	63 (10.7)^b^	84 (13.0)	89 (20.2)	0.001
Oxygen supplement	1 (1.7)^b^	27 (4.6)^b^	28 (4.3)	41 (9.3)	<0.001
IV fluid resuscitation	7 (11.7)	113 (19.3)	120 (18.5)	58 (13.2)	0.02
Intubation	0	1 (0.2)	1 (0.2)	2 (0.5)	0.36
Chest compression	0	0	0	0	
**Outcome**					
Biphasic reaction	0	9 (1.5)	9 (1.4)	6 (1.4)	0.97
Admission ≥24 h	26 (43.3)^b^	36 (6.1)^b^	62 (9.6)	157 (35.6)	<0.001
Prescription of epinephrine after discharge	6 (10.0)	43 (7.3)	49 (7.6)	6 (1.4)	<0.001
Death	0	0	0	0	

a Comparisons of proportions between the 2007–2016 and 2017–2024 groups.

b Statistical significance was observed when comparing pediatric and adult groups during the 2017–2024 period (p < 0.05).

The interval from symptom onset to ED presentation was significantly longer in the current cohort than in the previous study;^10^ the majority of patients arrived at the ED more than 1 h after the onset of symptoms (57.8% vs. 26.3%, *p < 0.001*). However, the mean time from ED arrival to the administration of the first dose of epinephrine was significantly shorter in the current period (10.3 vs. 16.3 min, *p* = *0.03*). Biphasic anaphylaxis was observed in 1.4% of cases, a rate consistent with our previous findings. Interestingly, hospital admission rates (defined as a stay >24 h) demonstrated a downward trend, particularly within the adult population. Despite the favorable acute outcomes at the CMU Hospital ED—evidenced by the absence of fatalities and only a single instance of respiratory failure requiring endotracheal intubation—the rate of self-injectable epinephrine prescription upon discharge remained notably low at 7.6%.

Out of 647 total episodes, 176 (27.2%) were classified as severe anaphylaxis. The potential risk factors and clinical variables associated with severe reactions are summarized in Table 7. Despite the high proportion of severe cases, multivariable logistic regression analysis did not identify any statistically significant factors independently associated with severe anaphylaxis in this cohort.

**Table 7:** Factors associated with severe anaphylaxis.

	Or (95%CI)	Adjusted OR (95%CI)^a^
Gender: Male	0.87 (0.52–1.22)	
History of allergic diseases	1.02 (0.64–1.33)	
History of asthma	0.95 (0.32–1.84)	
Underlying cardiovascular disease	1.02 (0.56–2.41)	1.45 (0.36–4.01)
Recurrent anaphylaxis	0.54 (0.23–1.06)	
Age <5 years	1.89 (0.89–2.91)	
Age >60 years	1.40 (0.78–1.86)	1.61 (0.91–2.91)
Causes: Food	0.87 (0.43–1.41)	
Insect stings	1.18 (0.67–1.87)	1.48 (0.36–1.61)
Drug	1.45 (0.76–2.12)	1.62 (0.75–2.01)
Time from onset to ER visit ≤30 min	0.99 (0.23–1.98)	

Severe anaphylaxis was categorized using the Brown Grading System (Grade 3), defined by immediate organ compromise manifest as hypoxia (<92% oxygen saturation on room air), cyanosis, hypotension, or loss of consciousness.

a Variables with p < 0.20 in univariate analysis were entered into the multivariable logistic regression model.

## Discussion

This 18-year retrospective analysis at a major tertiary center in Northern Thailand reveals a significant increase in anaphylaxis ED visit rate, rising from 93.8 to 288.3 per 100,000 ED visits, with adult presentations compounding by 10.17% annually (p < 0.01) since 2007. While the core hierarchy of triggers—led by food—remained consistent, we observed a distinct epidemiological transition in the specific allergens involved and an improvement in acute clinical management following the implementation of institutional best practice guidelines.

The consistent proportion of food-induced anaphylaxis (approximately 50%) over the 18-year study period reinforces global observations that food remains the primary driver of community-onset reactions, particularly in children. Shellfish persisted as the leading culprit across all age groups, mirroring the rising prevalence of seafood allergy recently documented in European cohorts.^15^ However, the internal composition of food triggers underwent a significant regional shift. The notable expanding proportion in reactions to shellfish and edible insects directly reflects the unique dietary and shifting landscape of Northern Thailand. Regional delicacies such as weaver ant larvae (*Oecophylla smaragdina*) and silkworms have transitioned from traditional foraged protein sources to prominent commercialized allergen categories in our cohort.^10^ This long-term surge is likely driven by the rapid commercialization and modern packaging of insect-derived foods, making them highly accessible in urban markets year-round rather than strictly on a seasonal basis. Furthermore, heightened diagnostic vigilance and more structured dietary history-taking introduced by our institutional protocols may have minimized historical underreporting. This emergence underscores the necessity for clinicians to incorporate local dietary habits into their diagnostic workup. Moreover, the high frequency of “unidentified edible insects' in our data suggests that patients may be reacting to diverse species available in local markets, which presents a significant challenge for precise allergen identification and subsequent prevention strategies. Crucially, the identification of a “complex food' subcategory (10.3%) highlights the practical clinical limitations of retrospective audits, where hidden multi-allergen ingredients in mixed local dishes frequently preclude the isolation of a single definitive trigger.

In contrast, the proportion of Hymenoptera-induced anaphylaxis decreased significantly compared to the 2007–2016 period. Northern Thailand was historically characterized by extensive commercial flowering orchards and agricultural lands where apiculture (beekeeping) was widely utilized for pollination.^16^ The observed decline in sting-related cases likely reflects the rapid urbanization of Chiang Mai, which has reduced the overlap between human habitats and nesting sites. Despite this downward trend, bees remain the most frequently identified insect trigger, likely due to the persistence of both traditional and commercial beekeeping in the peri-urban fringes of the city.

Our findings corroborate established literature suggesting that pediatric patients are more likely to present with respiratory symptoms and have a higher prevalence of comorbid asthma.^8^^,^^10^ Notably, cardiovascular manifestations—specifically tachycardia—were significantly more prevalent in the pediatric group (83.3%) than in adults. However, this finding warrants a cautious, dual-layered interpretation. First, pediatric tachycardia was carefully benchmarked against age-stratified physiological cut-offs to avoid the over ascertainment that occurs when applying static adult thresholds to young children. Second, tachycardia in a pediatric emergency setting is inherently non-specific and frequently reflects generalized pain, psychological distress, and procedural anxiety rather than representing a pure hemodynamic manifestation of systemic anaphylaxis.

Interestingly, we observed a higher prevalence of gastrointestinal symptoms and hypotension in the 2017–2024 cohort compared to the previous decade.^10^ While this might suggest an evolution in clinical severity or increased systemic involvement, a critical operational factor must be considered. After the 2017 implementation of revised institutional guidelines, all cases of suspected out-patient-onset anaphylaxis were mandated for management within the ED. The shift from outpatient clinic management to the ED environment likely resulted in more frequent vital sign assessments and continuous hemodynamic monitoring. Consequently, transient or early-stage hypotension may have been more accurately captured and recorded in the current cohort compared to the 2007–2016 period. Therefore, the observed “increase' in hypotension most likely represents a detection bias resulting from superior monitoring standards rather than a true shift in the physiological severity of the disease.

Evolving healthcare utilization patterns during the 18-year study window, most notably the profound structural shock of the COVID-19 pandemic, fundamentally shaped our epidemiological tracking. During the peak pandemic phase (2020–2021), the institution experienced a significant 20.13% contraction in baseline annual ED encounters across all diseases (*p* = *0.02*). Because overall ED patient volume shrank rapidly while acute, life-threatening emergency presentations like anaphylaxis remained static or decreased at a slower rate, the mathematically derived crude visitation rates per 100,000 encounters surged artificially during these years. This statistical artifact accentuates the importance of utilizing longitudinal Poisson models with population or institutional volume offsets rather than relying on unadjusted, year-on-year crude proportional comparisons.

The study demonstrates associations between standardized protocols and improved anaphylaxis management. The institutional guidelines implemented at CMU Hospital were closely aligned with the 2017 Thai National Clinical Practice Guidelines for Anaphylaxis.^17^ These protocols established clear diagnostic criteria for early recognition, utilized “easy-to-use' standardized order sets, and mandated prompt acute management within the ED. Beyond the clinical algorithm, our institutional approach included regular, structured training for ED care teams to ensure high-fidelity implementation of these protocols. The most encouraging result of these interventions was the significant improvement in acute management metrics. Intramuscular epinephrine administration rates reached 98.3%, and the mean time from ED arrival to the initial dose was reduced by 6 min (*p* = *0.03*). While a universal target threshold for epinephrine delivery is not explicitly standardized in the literature, this 6-min reduction may be clinically meaningful, as delayed epinephrine administration remains the primary driver of treatment refraction and fatal outcomes. However, because our design utilizes an uncontrolled, before-and-after observational framework, these improvements should be interpreted as associations rather than direct causal effects, as secular shifts in general clinical awareness, provider education, and structural ED staffing could act as unmeasured confounders. Furthermore, secular enhancements in record-keeping may introduce documentation bias that cuts both ways: the apparent 6-min treatment optimization and the documented increase in pre-hospital arrival delays (>1 h) may both partially reflect more meticulous electronic timestamping in the contemporary cohort.

A substantial proportion of our cohort (57.8%) experienced prolonged pre-hospital delays, arriving at the emergency department more than 1 h after symptom onset. These delays are primarily driven by a heavy reliance on private transport, low public emergency medical services (EMS) utilization, and under-recognition of early, non-cutaneous systemic symptoms. In contrast, reports from North America and Western Europe typically demonstrate faster median presentation times, backed by established pre-hospital EMS protocols and higher baseline deployment of epinephrine auto-injectors by patients or first responders.^7^ This prolonged care lag underscores an urgent need for community-level education focusing on early anaphylaxis recognition and prompt emergency activation.

Intriguingly, despite current international guidelines positioning intramuscular epinephrine as the singular first-line therapy and de-emphasizing routine adjunctive medications,^2^ our cohort demonstrated a significant upward trend in the co-administration of systemic corticosteroids (96.8%) and antihistamines (98.0%). This high rate of adjunctive therapy likely reflects entrenched physician prescribing habits, legacy institutional order sets, and defensive clinical ordering within a busy tertiary emergency department. While these agents may provide symptomatic relief for secondary cutaneous features (antihistamines) or address co-existing asthma exacerbations (corticosteroids), clinicians must remain cognizant that they do not substitute for prompt epinephrine administration nor reliably prevent biphasic anaphylaxis.^2^

Notably, zero anaphylaxis-related fatalities occurred during the study period. However, because baseline mortality from anaphylaxis is globally low, our study sample size was structurally underpowered to detect direct survival differences attributable solely to the protocol revision. Consequently, the absence of fatalities should be interpreted as a favorable clinical outcome within a broader framework of improved care delivery, rather than a definitive survival effect.

Despite excellent survival rates and zero mortality, the 7.6% prescription rate for self-injectable epinephrine at discharge remains a critical area for improvement. This low rate—frequently observed in middle-income countries—is likely driven by the high cost and limited accessibility of commercial auto-injectors in Thailand, as well as potential gaps in physician awareness regarding long-term recurrence risks.^18^^,^^19^ Given that 8.3% of our cohort had a history of prior anaphylaxis, the lack of accessible out-of-hospital epinephrine leaves a significant population vulnerable to future life-threatening events.^19^

Historically, epinephrine auto-injectors were not stocked in our hospital pharmacy. Consequently, only patients perceived to be at the highest risk for recurrence were prescribed epinephrine pre-filled syringes during follow-up consultations with an allergist, rather than at the point of ED discharge. To address this, following the implementation of institutional guidelines and in close collaboration with the hospital pharmacy department, a new protocol was established in 2024. Under this initiative, pharmacist-prepared adrenaline pre-filled syringes are now maintained in a state of constant readiness for immediate dispensing. By removing the barriers of cost and availability at the time of the emergency visit, we anticipate a significant increase in self-injectable epinephrine prescription rates upon ED discharge in future evaluations. The clinical and operational efficacy of this ongoing initiative represents a key direction for our future prospective tracking.

### Strengths and limitations

The primary strength of this study is its 18-year longitudinal scope, allowing for a robust analysis of shifting trends and the real-world impact of national guidelines. Manual adjudication and chart-by-chart validation by an allergist ensured diagnostic precision beyond administrative coding. The high volume of both pediatric and adult visits provides a robust sample size that reflects the diverse clinical landscape of Northern Thailand.

Despite its strengths, several limitations must be acknowledged. First, as a retrospective EMR-based study, our data are subject to the inherent constraints of clinical documentation quality and potential case under-ascertainment. While we utilized multiple ICD-10 codes for screening, true anaphylaxis cases that were completely miscoded as unrelated singular complaints (eg, isolated gastroenteritis or syncope without concurrent cutaneous manifestations) may have escaped detection, potentially introducing an ascertainment bias, Second, while the study identified a significant increase in hypotension and specific triggers, we cannot entirely rule out detection bias. The transition to the mandatory ED management protocol in 2017 led to more intensive monitoring and more frequent vital sign logging, which likely increased the capture rate of transient hemodynamic changes compared to the historical cohort. Third, confirmatory diagnostic testing—such as skin prick tests, serum-specific IgE, or oral food challenges—was only performed in a select subset of patients due to resource constraints and loss to follow-up. Consequently, the identification of certain triggers, particularly in “complex food' cases, multi-ingredient food exposures, relied primarily on clinical history. Furthermore, our institutional cohort may inherently underrepresent specific atypical or delayed-onset anaphylaxis phenotypes, such as alpha-gal syndrome (mammalian meat allergy) or food-dependent exercise-induced anaphylaxis (FDEIA). Because these syndromes often feature a protracted latency period of several hours between trigger exposure and systemic symptom onset, patients may fail to recognize the causal relationship or present to alternative ambulatory settings rather than the ED. Consequently, reliance on acute ED presentation records limits our capacity to capture the true longitudinal burden of these delayed hypersensitivity reactions within the region. Several population-specific limitations in children warrant consideration, including sample size constraints, non-specific vital sign fluctuations, and a primary reliance on caregiver-reported histories. Finally, as this study was conducted at a single tertiary referral center, the findings may primarily reflect urban and peri-urban patterns in Northern Thailand and may not be fully generalizable to primary care settings or other geographical regions.

## Conclusions

Our 18-year analysis highlights an increasing trend in ED visitation rates for anaphylaxis in Northern Thailand, characterized by a shift toward shellfish and edible insect triggers. Implementation of standardized protocols aligned with the 2017 Thai National Guidelines was associated with improved acute management, achieving near-universal epinephrine administration and zero mortality. To address the persistent gap in discharge prescriptions, our current pharmacy-led pre-filled syringe initiative offers a practical, scalable strategy to circumvent the high cost of auto-injectors in middle-income settings.

## Ethics statement

The study was conducted according to the guidelines of the Declaration of Helsinki, and approved by the Ethics Committee of the Faculty of Medicine, Chiang Mai University Hospital, Chiang Mai University. (protocol code PED-2568-0819 and date of approval December 12, 2025).

## Availability of data and materials

The datasets generated during the current study are available from the corresponding author upon reasonable request.

## Contributors

(i) Concept and Design: Pimchanok Rongdet, Pongsathorn Saligupta and Mongkol Lao-Araya (ii) Data collection: Pimchanok Rongdet (iii) Analysis and Interpretation of Data: Pimchanok Rongdet and Mongkol Lao-Araya (iv) Drafting the Article: Pimchanok Rongdet and Mongkol Lao-Araya (v) Final Approval of the Version to be Published: Pimchanok Rongdet, Pongsathorn Saligupta and Mongkol Lao-Araya.

## Disclosure of the use of generative AI and AI-assisted technologies

The authors used Gemini to refine the manuscript's language and flow. AI was not used for the creation of scientific, pedagogic, or medical content. The authors remain fully responsible for the accuracy of the clinical recommendations and scientific conclusions presented in this work.

## Conflicts of interest

The authors declare no conflict of interest.
